# Correction: Aquaro et al. Post-Mortem Cardiac Magnetic Resonance in Explanted Heart of Patients with Sudden Death. *Int. J. Environ. Res. Public Health* 2022, *19*, 13395

**DOI:** 10.3390/ijerph20095734

**Published:** 2023-05-06

**Authors:** Giovanni Donato Aquaro, Benedetta Guidi, Michele Emdin, Angela Pucci, Enrica Chiti, Alessandro Santurro, Matteo Scopetti, Federico Biondi, Aniello Maiese, Emanuela Turillazzi, Giovanni Camastra, Lorenzo Faggioni, Dania Cioni, Vittorio Fineschi, Emanuele Neri, Marco Di Paolo

**Affiliations:** 1 Academic Radiology, University of Pisa, 56126 Pisa, Italy; 2 ASL Toscana Nord-Ovest, 55100 Lucca, Italy; 3 Fondazione Toscana G. Monasterio, 56124 Pisa, Italy; 4 Scuola Superiore Sant’Anna, 56127 Pisa, Italy; 5 Department of Surgical, Clinical and Molecular Pathology and of Critical Area, University of Pisa, 56126 Pisa, Italy; 6 Department of Medicine, Surgery and Dentistry-Scuola Medica Salernitana, University of Salerno, 84084 Fisciano, Italy; 7 Department of Medical Surgical Sciences and Translational Medicine, Sapienza University of Rome, 00189 Rome, Italy; 8 Cardiology Department, University of Trieste, 34127 Trieste, Italy; 9 UO Medicina Legale, University of Pisa, 56126 Pisa, Italy; 10 Cardiac Department, Vannini Hospital Rome, 00177 Roma, Italy; 11 Department of Anatomical, Histological, Forensic and Orthopaedic Sciences, Sapienza University of Rome, 00185 Rome, Italy

The authors wish to make the following corrections to this paper [1]:

## Error in Figure

In the original publication, there was a mistake in Figure 6 as published. The picture on the right lower panel of Figure 6 does not belong to the clinical case depicted in the figure. The corrected Figure 6 appears below. 

The authors state that the scientific conclusions are unaffected. This correction was approved by the Academic Editor. The original publication has also been updated.

## Figures and Tables

**Figure 6:**
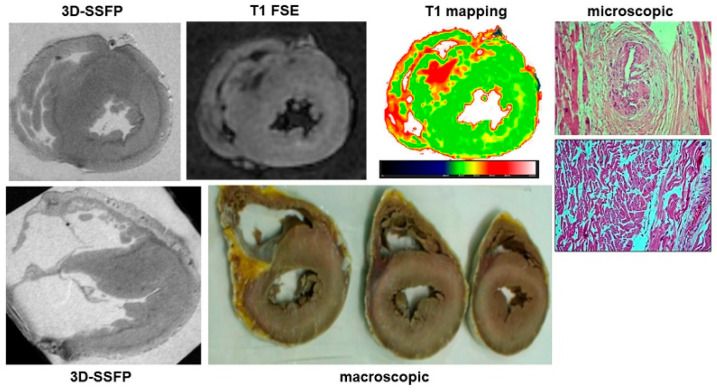
A case of hypertrophic cardiomyopathy. In 3D-SSFP sections, the asymmetrical hypertrophy of the interventricular septum is evident. In the same region, a large area of hypointensity in T1-FSE and increased T1 at mapping were found. Finally, myocardial disarray and perivascular and interstitial fibrosis were found at histology.
